# Selective visual processing across competition episodes: a theory of task-driven visual attention and working memory

**DOI:** 10.1098/rstb.2013.0060

**Published:** 2013-10-19

**Authors:** Werner X. Schneider

**Affiliations:** Department of Psychology, Neuro-Cognitive Psychology, Bielefeld University, PO Box 10 01 31, 33501 Bielefeld, Germany

**Keywords:** visual attention, visual working memory, biased competition, short-term consolidation, attentional blink, eye movements

## Abstract

The goal of this review is to introduce a theory of task-driven visual attention and working memory (TRAM). Based on a specific biased competition model, the ‘theory of visual attention’ (TVA) and its neural interpretation (NTVA), TRAM introduces the following assumption. First, selective visual processing over time is structured in competition episodes. Within an episode, that is, during its first two phases, a limited number of proto-objects are competitively encoded—modulated by the current task—in activation-based visual working memory (VWM). In processing phase 3, relevant VWM objects are transferred via a short-term consolidation into passive VWM. Second, each time attentional priorities change (e.g. after an eye movement), a new competition episode is initiated. Third, if a phase 3 VWM process (e.g. short-term consolidation) is not finished, whereas a new episode is called, a protective maintenance process allows its completion. After a VWM object change, its protective maintenance process is followed by an encapsulation of the VWM object causing attentional resource costs in trailing competition episodes. Viewed from this perspective, a new explanation of key findings of the attentional blink will be offered. Finally, a new suggestion will be made as to how VWM items might interact with visual search processes.

## 1. Visual attention and visual working memory: a biased competition approach

### (a) A brief sketch of the biased competition framework for understanding visual attention

Humans sample visual information from their current environment by successions of discrete sampling episodes, the so-called fixations, which are interrupted by fast ballistic eye movements, the saccades [1]. The extraction of useful visual information is restricted to periods of fixations. Within a fixation, visual processing is capacity limited, that is, only part of the information simultaneously present at the retina is available for perception (e.g. verbal report), sensorimotor action or encoding into long-term memory. The *biased competition* framework ([2]; see also [3–6]) describes how these capacity limitations might emerge. Competition in visual processing means that neural representations of objects and events are characterized by limited capacity on the one hand and its counterpart, selectivity, on the other hand. Only a few of these representations (e.g. of visual objects) can be ‘used’ simultaneously, for example, for report or spatial sensorimotor actions. Bias implies that selection among competing representations does not occur on a random basis. Instead, selection is guided by top-down factors such as the current task and by bottom-up factors such as the ‘saliency’ (intrinsic quality) of a stimulus representation.

Some biased competition theories [3] assume that top-down and bottom-up factors of attentional control are combined within a ‘priority map’ ([7,8] but see [9] for arguments against such specialized maps for attentional control). A priority map computes for each location (e.g. of an object) attentional priorities. On the basis of these priorities, attentional control signals are sent back in a location-specific manner to visual feature maps, that is, they bias competition there. As a consequence of these control signals from a priority map certain objects and their visual features win the competition over others and can therefore be accessed by goal-directed actions such as report. The existence of such a priority map for attentional control—sometimes also called saliency map—is also implied by non-biased competition theories of visual attention that assume the serial allocation of attentional resources in space [10,11]. Single cell recordings in non-human primates and other findings suggest that a subset of neurons of frontal eye field (FEF) [12], lateral intraparietal area (LIP) [8], superior colliculus (SC) [13] and the pulvinar [14] might be involved in creating a priority map. A central open question is how these neurons of various primate brain areas build a common functional priority map in order allow coherent selection processes and behaviour [15].

In summary, the biased competition framework claims that ‘at some point (or several points) between input and response, objects in the visual input compete for representation, analysis or control. The competition is biased, however, towards information that is currently relevant to behaviour. Attended stimuli make demands on processing capacity, whereas unattended ones often do not’ [2, p. 194]. From this point of view, various experimental phenomena of visual attention (e.g. findings from the partial report or visual search task) are viewed as an emergent property of biased competition [9].

How might priority computation be used for biasing competition in visual processing? Two main classes of visual attention theories can be distinguished (see [16]). The first class, the so-called *serial visual attention theories* [11,17], assumes that only one location (coherent region) and/or one object of the priority map at a time sends out in a location-specific manner attentional signals (e.g. in the form of a spotlight; [18]) to visual feature maps. Consequently, these serially attended visual features can be used for the task at hand (e.g. for deciding whether a T is present in a visual search task). Moreover, serial models assume sometimes that the currently attended location comprises only one object, so that fast attentional scanning one object after another (e.g. every 50 ms) should occur [11]. The second alternative class of visual attention theories [3,19,20] is called ‘*parallel-and-capacity-limited theories*’*.* They imply not only the computation of attentional priorities (e.g. attentional weights; [19]) for each location and/or object, but also assume that these priority values are normalized in a capacity-limited manner (e.g. in the form of relative attentional weights). Based on these normalized priority values, an attentional output signal is sent in a location-specific manner to visual features in the corresponding cortical maps (e.g. V4 or middle temporal area (MT) [3,19]). The higher the priority (e.g. attentional weight) of the output signal for visual features of location and/or object, the more attentional resources are allocated there, and the higher the chance that the features at this location will win the competition for being used in perception, memory or sensorimotor action [3,19]. As stated above, and in line with the biased competition framework, the overall amount of visual attentional resources that are distributed within a fixation is capacity-limited. Therefore, the more resources are allocated to one object, the less resources are available for the processing of other objects [3,19,20].

### (b) The ‘theory of visual attention’: a specific version of the biased competition framework and its neural interpretation

The approach, taken, in this review, for understanding task-driven competitive visual processing, is based on a specific parallel-and-capacity-limited theory of visual attention (TVA), developed by Bundesen [19], namely the ‘TVA’ and its recent neurophysiological specification, namely a ‘neural theory of visual attention (NTVA; [3]). TVA and NTVA can be viewed as specifications of the biased competition framework in terms of computational theories [21,22]. They describe task-driven selective visual processing within an eye fixation. TVA explains within a formal mathematical language a large dataset from classical experimental paradigms of attention research such as visual search, partial report or spatial cueing [16,19]. NTVA delivers a specific neural interpretation of TVA and it explains major single cell recording results of attentional manipulations at the level of cortical neurons such as V4, inferior temporal cortex (IT) or MT [3].

TVA implies a competitive race of sensory visual information towards visual short-term memory (VSTM). VSTM information can be used for task-driven actions such as partial report or deciding whether a target object was present in a visual search display. In NTVA [3,21], this competitive race towards VSTM is divided into two successive phases (waves) of visual processing. During the unselective *phase 1*, attentional weights are computed within a priority map. The weights are computed for early visual object representations and they are assumed to be bias object-based competition for VSTM access in *phase 2* of visual processing. More precisely, this selective second phase consists of a weight-guided race (competition) of visual objects via their features towards capacity-limited VSTM. Importantly, the higher the *attentional weight* of an early visual object representation, the better its chance that one of its visual features reaches capacity-limited VSTM in time, that is, before all VSTM slots (a limit of about three to four) are taken or before their visual input is replaced (e.g. in backward masking). In this review, these early visual object representations with attentional weights in priority maps and visual features in cortical maps are called proto-objects [23–25]. The term proto-object should make explicit that these early representations of visual objects cannot be used for goal-driven actions such as report (‘access consciousness’; [16]). In other words, transformation (further processing) of attentionally selected, competition winning proto-objects into VSTM objects is necessary for goal-directed actions.

Besides computing and applying attentional weights by the process of ‘filtering’, TVA and NTVA assume a second attentional process, called ‘pigeonholing’ (see [19]). It can be considered as ‘response category related’ and acts in a spatially unspecific manner after priority-based attentional modulation (see [21]). Both types of visual attentional processes determine jointly the competition winners that are encoded into VSTM. For phenomena discussed in this review (e.g. saccade target selection or the attentional blink, AB), only the ‘filtering’ process is relevant; therefore, the ‘pigeonholing’ process will not be included in the following considerations. In NTVA, encoding in VSTM is specified as setting up a *loop* between visual features of an object and its object node within a ‘VSTM map of objects’. As soon as a feature of an object is encoded into the VSTM map of objects, a VSTM slot is reserved for other features of the same object. A slot-based limit of VSTM of about three to four objects is assumed [19].

As stated above, TVA and NTVA imply a distinction between successive form*s of visual object representations* within the visual mind and brain of human and non-human primates. The first form for representing an external visual object refers to visual features/visual categories that are segmented into elementary visual object representations [16,19]. Here, these early object representations that are not accessible for goal-directed actions are called proto-objects*.* The following characteristics are ascribed to these proto-objects. First, following Wischnewski *et al*. [25,26], proto-objects can be broken into two parts. The first part of a proto-object refers to its visual features/categories within the ventral and dorsal stream (e.g. maps of V1, V2, V4, MT, etc.). The second part of a proto-object is represented within a *priority map* [7]) and refers to an early spatially extended representation of a tentative external object—here called a *priority map region*. Each priority map region is temporarily connected to the visual features in cortical maps. Besides retinal location within the priority map, the region has a rough shape [25,26] and, importantly, an *attentional weight* [19]. Following TVA, the weight of a proto-object region within a priority map modulates, in turn, the race of its temporarily linked visual features in cortical maps towards VSTM, that is, it influences the competition of visual features of the proto-object for VSTM access. One might say that proto-objects compete for VSTM access.

Once features of proto-objects are encoded into VSTM, a second form of visual object representation emerges. Here, these representations are simply called *visual working memory* (*VWM*) *objects*—visual tokens [27] or object files [28,29] might also be proper names. In other words, after VWM encoding, a proto-object is converted into a VWM object. Only VWM objects can be used for goal-directed actions. For young adults, up to about three to four visual objects can be encoded and maintained within VWM (see [30] for evidence on ‘magical number four’, and [31] for a recent review of evidence for the slot-based nature of VWM). As stated above, NTVA specifies VWM objects in terms of loops between a ‘VWM map (VSTM map) of objects’ and its visual features in cortical maps (e.g. V4, MT). The object representation node within the VWM map of objects can be seen as a pointer to visual features (see [3]). At the neurophysiological level, VWM objects could be coded by synchronized and phase segregated activity patterns [27,32,33] of the distributed representations of visual features and object pointers. If so, then synchrony-based binding could be realized via these object pointers—binding should be the result of attentional selection in processing phase 2, more precisely phase 2 [27,34].

Moreover, the activation-based VSTM conception of NTVA includes visual features within cortical maps (e.g. V4, MT) as part of the VSTM process, as part of the loop. Therefore, visual perception and VWM cannot be structurally and functionally completely segregated. They should rely, in part, on overlapping structures and representations such as visual features as parts of online perception and of VWM (for a summary of confirmative experimental evidence about this claim of shared visual attention (in perception) and VWM processes, see [35,36]). In summary, two successively computed forms of representing an external object (stimulus) within the visual mind and brain are distinguished here, namely proto-objects and VWM objects. While proto-objects include all visual objects that can be extracted from the current fixation, the number of VWM objects is more limited and does usually not exceed the limit of three to four objects.

### (c) Visual selection with eye movements: ‘where to look next?’ and its coupling to visual attention for perception

Natural vision proceeds as a succession of fixations and intervening saccades. The primate retina with its inhomogeneous structure has a much higher resolution in the centre, the fovea, than in the periphery [25]. This anatomical fact can be seen as one reason for the high occurrence of saccadic eye movements (three to four times per second) to potential informative retinal locations (e.g. informative for the current task; [37]). Given a biased competition framework, the question emerges of how the next saccade goal (‘where to look next?’) might be selected. Currently available data of numerous studies of the past two decades deliver a converging answer, namely that a goal-directed, task-driven saccade to a location in space has to be preceded by the allocation of covert visual attention to this location (for an non-attentional perspective to saccade control, see [38,39]). Deubel & Schneider performed—inspired by the visual attention model (VAM; [27])—a series of experiments on this issue [40–43]. Their findings demonstrate that the preparation of a space-based sensorimotor action such as an eye movement (or even an arm movement) to a target object binds simultaneously attentional resources for perceptual analysis to this location. For example, Deubel & Schneider's [40] study required participants to make a saccade to a trial-wise cued saccade target object among other distractor objects. Prior to the saccade execution, that is, during the preparation phase of the eye movement, a perceptual discrimination target (DT) was briefly flashed at the location of saccade target, or at the location of nearby objects (pre-mask elements just one degree away). The data show clearly best perceptual performance when saccade target and DT refer to the same object. If they are just a degree apart, DT performance dropped substantially. Based on these and other studies [44–46], it can be concluded that biasing competition for *saccade target selection* (selection-for-action) and biasing competition for *perceptual report* of a target (selection-for-perception) are *strongly coupled* to one common target [27,43] or even to several targets [47].

These findings on couplings of competitive visual processing for perception and for action are beyond the scope of TVA and NTVA in its current form. These theories explain behavioural and neurophysiological results of selective perception tasks such as visual search but do not make any claims of how selection-for-spatial-motor-action [15,27,48] might work and how it might be linked to selective perception. However, for understanding biased competition dynamics in visual attention and VWM beyond a single fixation, it is necessary to state how the function of saccade target selection, of ‘where to look next?’ might be handled within the biased competition framework. Following earlier work from my laboratory [25,26,49] and compatible with VAM [27], it is assumed that ‘where to look next?’ (saccade target selection) should be based on the *attentional weights of TVA*. During the preparation phase of a saccade and just prior to movement execution, the *priority map region* with the currently highest attentional weight should serve as the next saccade target. This suggestion implies that priority map regions couple selection-for-perception (covert visual attention) with selection-of-saccade-target-location. As a consequence of priority map modulation by attentional weights, up to four proto-objects (their visual features) win the competition for VWM access in perception and, simultaneously, one proto-object with the highest attentional weight determines ‘where to look next’. In summary, a covert attentional weight-guided biased competition process should not only modulate access of proto-objects to perceptual VWM, but should also determine—mediated by the same priority map regions—the target location of the next saccade.

## 2. Visual working memory: beyond encoding

### (a) Phase 3 of visual processing: the task-driven use of visual working memory information

What is biased competition in visual processing for? It should make visual information *available for goal-directed behaviour* such as space-based sensorimotor actions or verbal report actions [2,9,19]. One interesting case of goal-directed behaviour refers to *actions* controlled by the *current task* [15,37,50]. How are results of biased competition of visual processing made available for performing actions in the service of the current task? As stated before, in the specific versions of the biased competition framework such as TVA [19] and other attentional theories [20], it is assumed that *capacity-limited VSTM*, here labelled VWM, should represent the results of biased competition. This suggestion implies that encoding visual information into VSTM makes it available for goal-directed behaviour and cognition. NTVA [3] conceptualizes VSTM as reverberating loops between visual features/categories and a node of slot-limited ‘VSTM map of object’. As long as visual information is part of such an activation-based loop, it can be used for behaviour (e.g. grasping) and cognition (e.g. recoding into verbal format). An interesting implication of NTVA is that these loops of up to four objects are not only used for short-term retention, but they should also form the basis for online perception of stimuli that are present at the retina during current fixation. Supportive evidence for this claim comes from a recent study of Tsubomi *et al*. [51] showing that continuously visible and no longer visible objects share the same capacity limit and neural signature of VSTM (e.g. contralateral delay activity; see [52]).

Extending the two visual processing phases, postulated by TVA and NTVA, the *use of VWM/VSTM information for task-driven behaviour and cognition* should be labelled *phase 3 of competitive visual processing*. ‘Use of VWM information’ can mean to initiate an already prepared sensorimotor action or store visual information for the delayed use in later competition episodes—examples will be given in the next subsections. It is suggested that only currently relevant VWM objects should be subject to the third phase of visual processing. For instance, if a non-relevant object is encoded into VWM owing to high visual similarity to a currently relevant object (e.g. a distractor in an inefficient feature search task might make it into VWM; see [19,20]), then this non-relevant object should have a low probability of being processed further in phase 3. Given that a key function of VSTM is to make visual information available for goal-directed behaviour and cognition, for phase 3 operations, the term *VWM* seems to be more appropriate than the term VSTM chosen by TVA and NTVA. The term working memory implies that storage—a key facet of VSTM—is just one function of this computational structure. Visual processing of encoded VSTM information for its task-driven use in behaviour and cognition should be the other important function (such as ‘manipulation’ of visual information; [53]).

In summary, the use of VWM objects for task-driven behaviour and cognition is the key function of phase 3 processing. Understandably, standard theories of visual attention such as TVA, NTVA, feature integration theory [34,54], guided search [11] or Itti *et al*.'s [17] computational model of visual attention have little to say how phase 3 processing might work. Their main focus is on how selection of visual information for further processing such as object recognition, working memory encoding, etc. works. Given that visual information processing capabilities of primates evolved in the service of efficient goal-directed behaviour [15,27,48,50,55], the issue of the *use of VWM information* should be of central importance for understanding vision as a whole. The most recent extension of TVA, namely the ‘theory of temporal visual attention’ (TTVA) by Petersen *et al.* [56], suggests an interesting answer to the question of using visual information. TTVA states that ‘recoding’ of VWM information into ‘nonvisual (e.g. auditory, motoric or amodal) format’ is the next step of dealing with VSTM objects. *Recoding* should allow, for instance, verbal report of a VSTM object or a motor response based on a VSTM object (e.g. grasping).

In the next section, one form of using VWM information, namely short-term consolidation will be the focus of interest. It is a process that generates passive VWM representations that do not suffer from attentional resource costs of active VWM maintenance. Another class of phase 3 processes refers to the use of active VWM information for immediate sensorimotor actions. Action targets in space might already been specified at the level of proto-objects within activity-specific priority maps (e.g. neurons in FEF or SC for eye movements or even neurons in the anterior intraparietal area for hand movements)—‘where act next?’ is settled there. However, for triggering such a prepared motor action, it is suggested that VWM encoding of task-relevant features of the action target must have taken place. A goal-directed action should only be initiated if its triggering conditions are given (if condition ‘X’, then action ‘Y’). In other words, phase 3 of VWM should be necessary in order to allow ‘action initiation’.

### (b) Passive visual working memory: retaining visual information for task-driven actions across several fixations without attentional resource costs

An informative constraint on how VWM information could be used will now be derived from findings of how human and non-human primates sample and retain visual information over time in the service of the current task. Sampling occurs in permanently ongoing successions of fixations and saccades, that is, usually three to four saccades per second are executed [1,57]. Therefore, on average, 250–300 ms of online processing time within a fixation are available for visual computations from phases 1 to 3. Is VWM information always used within the current fixation? No. Human and non-human primates can perform task-driven actions on visual information that has been sampled in immediately preceding fixations. First, there is ample evidence that a limited amount of visual information can be retained across one or several saccades, namely information about four visual objects [29,58]. This short-term form of transsaccadic retention of visual objects might possibly be supplemented by ‘visual long-term memory components’ (passive VWM?) that results in a moderate transsaccadic memory performance increase [59]. These findings from transsaccadic memory studies led to the conclusion that the *retention of visual information across saccades* for the current task relies at least in part on *VWM* with a capacity-limit of about four objects [29,58,59]. Moreover, transsaccadic retention of visual objects can be influenced by their task relevance within individual fixations. More relevant items presented within some of the successive fixations can be prioritized and therefore be recalled and recognized better later on [60].

Second, besides findings from these just mentioned highly controlled laboratory tasks, further important constraints on the transsaccadic use of VWM for task-driven action control should now be derived from studies of *real-world tasks* [1,61–63]. These tasks usually require using visual information from more than a single fixation. This is especially valid in dynamic environments of fast sport games (see [1]) whose task performance has sometimes been based on visual information sampled across several saccadic eye movements. Sometimes, task-relevant visual information is extracted within one fixation and is used a number of fixations later. Imagine, for instance, a football (soccer) player who wants to pass to a team-mate surrounded by several opposing defenders. The first saccade of the player may go to the current position of this team-mate, and the next saccade may go to the defenders. Finally, the player may saccade to the probable future position of the team-mate for planning the pass by using the previously sampled information (e.g. about the defenders). Moreover, the functional analysis of the everyday task of ‘sandwich making’ studied by Hayhoe & Ballard [63] should make the requirements of task-driven short-term retention for later task steps even clearer. Imagine, you are sitting for the first time in front of the kitchen table of our friend and were asked to make a peanut butter sandwich. Your friend has put all the necessary ingredients already on the table. You will probably first scan the table with your eyes in order to acquire knowledge about which object is where. During the task of making the peanut butter sandwich, later steps such as ‘grasping the jelly glass’ should rely on previous sampled information about location and identity of relevant objects (jelly glass). Therefore, it seems unlikely that a ‘visual search without memory’ is performed in every task step of such multi-step everyday activities (see also [1]). Therefore, short-term retention about information sampled in previous task steps for later use might be an advantageous strategy for efficient task control.

In summary, empirical evidence from transsaccadic memory studies and functional considerations about sport tasks and everyday activities suggest that task-relevant visual information sampled in preceding fixations can be retained at the short-term scale and used in later fixations for the current task step. Combining this observation of task-driven short-term retention with the biased competition architecture of TVA and NTVA, a *retention-encoding dilemma of VWM* is postulated. On the one hand, VWM should be able to retain a limited number of task-relevant visual objects (within its three to four slots) over the course of several fixations for later use in the current fixation. On the other hand, task-driven competitive visual processing in each new fixation requires a VWM that has ‘space’, has slots available for encoding the competition winners.

An evident solution for handling this retention-encoding dilemma assumes, on the one hand, the selective retention of task-relevant visual information within some of the VWM slots and, on the other hand the selective clearance of other VWM slots prior to each fixation so that ‘space’ for new competition winners is made available. The existence of a clearance process for VWM (VSTM) has been explicitly postulated by NTVA [3] prior to the start of a new race (competition); moreover, Duncan & Humphreys’ [20] visual attention theory claimed that VSTM is cleared at the start of new fixation. Clearance of VWM creates room for new winners of the race, for a new phase of competitive visual processing. Therefore, relevant items for the current task should be maintained across fixations while non-relevant items that may have also won the competition (e.g. in the cases of high target distractor similarity, see [19,20]) should be eliminated from VWM. However, this suggestion of *selective maintenance/selective clearance of objects within VWM* comes with substantial *costs in terms of attentional capacity*.

Actively maintained winners from preceding fixations reduce the number of limited slots for competition winners of the current fixation. Moreover, these maintained winners touch a second visual processing capacity limit, namely in terms of normalized attentional weights. Each maintained previous competition winner has an attentional weight that competes via normalization with other attentional weights of the current fixation. NTVA [3] states that maintenance of a visual object within VWM should be activation-based implying that visual features within perceptual brain areas are maintained (e.g. ventral and dorsal stream areas such as V4, IT and MT; for a review, see [36]). Activation-based maintenance implies that not only features but also attentional weights—crucial for competition—are retained. In other words, the activated and maintained visual features of a VWM object, in turn, should feed into a corresponding attentional weight that also is maintained. Therefore, top-down bound *attentional weights of actively retained VWM objects* from preceding fixations act as *further competitors within the* biased competition process of the current fixation. Attentional weights of all online items of the current fixation should compete via their attentional weights with maintained items from preceding fixations. In other words, it implies that actively retained VWM objects bind attentional weights from previous fixations. Therefore, the competitive processing of visual information within the current fixation should be substantially slowed down by maintained visual objects from preceding fixations. Therefore, this activation-based transsaccadic form of VWM maintenance implies *ongoing attentional costs during the maintenance phase*.

### (c) Passive visual working memory without attentional resource costs, short-term consolidation and retrieval into the active form of visual working memory

How might these ongoing attentional costs of selective activation-based VWM maintenance—blocking VWM slots and ongoing binding of attentional resources (attentional weights) during retention—be avoided? It is suggested that a *further form of VWM retention without permanent attentional resource costs* exists in the primate brain and that this form of short-term retention is realized by *passive VWM.* On the basis of a variety of findings and on computational considerations, a number of authors [64–66] argued for the existence of such a passive VWM based on very short-term synaptic changes. Decisively, passive VWM traces of visual objects would not lead to attentional resource costs in terms of occupying slots in active VWM and binding attentional weights as further competitors.

How could such a passive VWM be created? Following Hebb's [67] suggestion for long-term memory encoding, the generation of a passive VWM representation might presuppose the retention of *objects within activation-based VWM*. Here, the encoding of an object into activation-based VWM implies that the loops between visual features and its pointer within the ‘map of VWM objects’ are set up [3]. Such object-based VWM loops should be necessary for a transfer of the activation-based code into a passive code that might rest on short-term synaptic changes [66,68]. Borrowing a term from Jolicoeur & Dell'Acqua [69], this transfer of a visual object from active to passive VWM should be called ‘short-term consolidation’*.* The duration of such a consolidation process could follow an exponential distribution [69] and it might be modulated by parameters such as importance or arousal. What is the difference between short-term consolidation and VWM encoding? *Encoding* into VWM means setting up the activation-based loop for a visual object, whereas *consolidation* means creating a passive code within VWM as a result of a sufficient looping duration. In summary, a distinction is suggested between *activation-based VWM* and *passive VWM*. Short-term consolidation refers to the process of transferring relevant visual objects from active VWM into passive VWM.

Evident questions for this conception of active and passive VWM are: how might the limit of three to four objects emerge? How might retrieval from VWM work? Following NTVA [3], it is assumed that the capacity limit of activation-based VWM is the result of a *k*-winner-take-all process between object pointers of the VWM map given *k* might be three to four for young adults. Therefore, within one competition episode of a single fixation, only *k* winners can be subject to short-term consolidation in phase 3. If so, then many competition winners (many active VWM objects) could be stored across several fixations in passive VWM—much more than *k* winners of one competition episode of one fixation. Given this analysis, why does a limit of three to four objects emerge? At the beginning of the retrieval process, the *k*-winner-take-all network of active VWM should be initialized (set to zero). An external signal might start competition between passive VWM objects and only three to four winners can emerge within the activation-based VWM map of objects.

How might retrieval from passive VWM be controlled? Importantly, the storage in passive VWM serves the key function of allowing task-driven actions based on objects encoded in preceding fixations, preceding competition episodes. Therefore, a key factor that influences the chance of retrieval competition should come from the current task step. For instance, the task ‘making of a peanut butter sandwich’ requires as one step ‘grasping the jelly glass’. Therefore, during this step, the position of the jelly glass should be retrieved from passive VWM for allowing an eye movement to the jelly glass and a following grasping movement (for eye-hand sequence coordination; see [70,62]). Long-term memory knowledge might restrict where the glass might be (e.g. on the table) but its position relative to other objects (for this specific table at this point in time) should be a matter of passive VWM. The pointer of the currently task-relevant object within passive VWM (e.g. pointer for the jelly glass) might be activated during retrieval by the triggering condition of the current task step (‘grasping of the jelly glass’). Further context factors for retrieval besides the current task step could be a representation of the current scene (e.g. in the sandwich example, the kitchen scene or in VWM experiments the current trial within a certain laboratory context with screen, etc.). In summary, the current task step and the current scene might be major factors in determining which objects of passive VWM will win the competition for becoming again a member in activation-based VWM. Evidently, various new experimental studies are required in order to test whether passive VWM actually exists and how shot-term consolidation and retrieval from such as passive system might work—for a review of experimental studies on retrieval from VWM, see Gazzaley & Nobre [36].

Cowan [71] and Oberauer *et al*. [72] put forward influential domain-unspecific models of working memory. Their models view working memory processes as an activated part of passive long-term memory. How is this approach different from task-driven visual attention and working memory (TRAM)'s assumption of passive VWM? First, Cowan [71] and Oberauer *et al*. [72] argue for a central capacity limitation. TRAM, instead argues for different forms of capacity limitations even within the visual modality (e.g. normalized attentional weights within priority maps, a slot limit of about three to four objects by a *k*-winner-take-all VWM map of objects) that should not be reduced to one common limitation. Second, TRAM has a more limited explanatory goal in terms of domains and phenomena. It focuses on competitive visual processing and relies on middle-range theories of visual attention and VWM (NTVA) that attempt to explain findings from vision studies such as visual search or partial report. Third, according to the viewpoint taken here, terms such as ‘focus of attention’ within working memory [71,72] imply the risk of inadequately mixing different processes such as visual attention for modulating access to VWM (e.g. filtering in TVA) or task-driven maintenance processes within VWM into one common construct. Fourth, TRAM offers a process theory that specifies processing events by postulating that competitive VWM encoding (phases 1 and 2) is followed by the ‘use’ of VWM information (phase 3). Such a process account is not the explanatory goal of Cowan's [71] or Oberauer *et al.*'s [72] versions of central capacity theories of working memory. Instead, informative data on individual differences in complex working memory tasks are the ‘explanatory targets’ of these theories (see also [31]).

## 3. Visual working memory processing (phase 3) across competition episodes

An interim summary of the theory of TRAM specified so far should be given. At the start of a fixation, phase 1 of visual processing computes visual proto-objects that consist of visual features at different levels of the cortical hierarchy and priority map regions. Each priority map region receives an attentional weight, a measure of attentional priority. Each weight is based on stimulus-driven factors such as sensory evidence for its features and on top-down factors such as ‘pertinence’ (e.g. importance of object for the current task). At the end of processing phase 1, after the computation of relative weights, VWM should be cleared in order to create ‘space’ for encoding new competition winners. At the start of phase 2, visual feature processing is competitively modulated by the normalized weights, the attentional priority settings computed in phase 1. Weight-based feature modulation means that competition between proto-objects is biased. Those proto-objects, their features with higher attentional weights will have a higher chance of winning the competition for being encoded into capacity-limited active VWM than proto-objects with lower attentional weights. For young adults, three to four objects can be encoded in active VWM. Importantly, competition between proto-objects is regulated on the basis of priority-(weight)-modulated features. The faster the ‘race speed’ of a feature, the higher its competition value, the higher its chance to access a VWM slot before all slots are taken. If a feature of a proto-object is encoded into VWM (given there were still free slots), then this feature occupies the slot for all other later competition winning (later arriving) features of the same proto-object. Once features of proto-objects are encoded into VWM, a second form of object representation emerges, namely VWM objects, and phase 3 of visual processing starts for relevant objects (that match, e.g. the task set). One phase 3 operation is short-term consolidation that transfers the activation-based code (loops between a VWM map and features) into a passive code that might rely on short-term synaptic changes. A key advantage of passively retained VWM objects is the missing attentional resource costs in terms of slots and attentional weights.

This sketch of TRAM has an important implication. A new competition episode generates at the end of phase 1 a clearance signal for active VWM in order to achieve ‘space’ for encoding new competition winners [3,20]. Phase 3 of visual processing that uses VWM information for behaviour and cognition should be finished before a new competition episode, a new race starts. What happens if phase 3 of the current competition episode, for example, short-term consolidation, is not finished while a new episode is called by changes in visual stimulation? An informative example is given by a standard backward masking experiment [73,74]. A briefly presented and pattern-masked target stimulus should be reported without time pressure. Given a brief stimulus onset asynchrony (SOA) between target stimulus and mask (e.g. 80 ms), it seems rather likely that phase 3, short-term consolidation, could not be finished prior to mask appearance. The mask calls a new competition episode—attentional weight settings have been changed from the target to the mask object (see below). What allows short-term consolidation of the target element to be finished despite a call of a new competition episode by the mask and therefore a call of VWM clearance? The following two subsections will suggest an answer in terms of a *protective maintenance process*. In short, the protective maintenance process allows finishing phase 3 operations on VWM objects during subsequent competition episodes. Protective maintenance simply protects the VWM slot from being cleared. As stated in §2, such non-clearance of VWM should have substantial attentional costs in terms of slots and attentional weights. In the following sections, an analysis of key findings from the ‘AB’ paradigm [75–77] will reveal crucial aspects about this type of attentional resource cost. According to TRAM, these costs—as measured within the second target deficit within the AB paradigm—should reflect competition between protectively maintained and encapsulated VWM objects (encoded in preceding competition episodes with unfinished phase 3 operations), on the one hand, and proto-objects of the current episode (that attempt to access VWM) on the other hand.

### (a) Discrete visual processing over time: defining competition episodes

The term ‘competition episode’ has been used in the preceding paragraphs. Now, a more formal definition will be given following TVAs [19] idea about a race. A new race during fixation should be called if the relative attentional weights change. Bundesen [19, p. 536] discusses this case under the label of ‘many view search’. A change in relative attentional weights, a change in attentional priority, should lead to a new race, a new competition episode. Therefore, a new competition episode will be called if the relative attentional weights change. Relative attentional weights are computed as the result of a normalization process of all absolute attentional weights. Relative weights refer to all proto-objects of the current race, the current competition episode. Not every change of visual input leads to a change of relative attentional weights and to a new competition episode. For instance, if further features/categorizations of a proto-object are computed as stimulus quality improves over time, then this change of visual input should not cause a change of attentional priorities and the competition episode should go on.

A new competition episode can be triggered internally, by a new task step implying a shift of the attentional set, or it can be triggered externally, by visual input accompanied with weight changes. An example of an internal triggering by an attentional set shift can be illustrated with the ‘peanut butter sandwich making’ example. If the task step ‘grasping the jelly glass’ is finished, then the next step of ‘transporting the glass to the desired location’ should be initiated. For the first step, the current location of the jelly glass is relevant, whereas for the following step, the future location of the glass is relevant and should therefore be part of the changed attentional set. An externally triggered new competition episode occurs if changes in visual stimulation are accompanied by changes in relative attentional weights. If an object is moving into (or out of) the retina, then the new weight (or disappearing weight) of the moving object should lead to recomputation of relative weights. Moreover, if objects within a fixation are *occluded*—either owing to movements of the occluder and/or of the occluded object [78]—then relative attentional weights have also to be recomputed.

### (b) Protective maintenance of short-term consolidation during subsequent competition episodes. I. Encapsulation of visual working memory objects and attentional resource costs

Given our definition of competition episodes, the key question of §3 can be tackled: what happens if *phase 3* processing of a competition episode is still going on and is *not finished* while a new competition episode is triggered by changes of attentional priorities? Is VWM cleared completely so that new competition winners can be encoded? An advantage of this solution would be to have no attentional resource costs (see the arguments in the section ‘visual working memory: beyond encoding’). However, such complete VWM clearance would have remarkable costs, namely the use of VWM information in phase 3 for behaviour and cognition would be interrupted. This would imply loosing information from VWM without any chance of recovery. However, as sketched above, even in a simple backward masking experiment with short SOAs with likely unfinished short-term consolidation, targets can be reported clearly above chance level. How is this possible? The suggested answer is a *protective maintenance process.* A protective maintenance process allows finishing phase 3 operations for a VWM object (e.g. such as short-term consolidation) during subsequent competition episodes. Protective maintenance simply prevents that an active VWM slot with an ongoing phase 3 operation is cleared at the start of a new competition episode. Importantly, it is selective protection of only those slots in which phase 3's short-term consolidation process is still going on. During phase 1 of a new competition episode either updating or encapsulation of a VWM object should occur. On the one hand, *updating* is issued if a VWM object receives visual input that fits in terms of its priority map region characteristics (location, rough region shape and attentional weight) to the predicted (expected) region characteristics maintained by the VWM object. In other words, updating is called if the visual system signals for new visual input to a VWM object (e.g., after a saccade) object continuity. On the other hand, if the new visual input signals a failure of object continuity of a VWM object then *encapsulation* should take place. It is suggested that encapsulation implies that the visual features and the attentional weight of the encapsulated object are retained at the current state (current activation level) at the moment of the encapsulation call. Crucially, an abrupt change in terms of the priority map region's expected location, rough shape or attentional weight should trigger encapsulation.

The ‘TTVA’ [56] made a similar suggestion as the protective maintenance process introduced here. TTVA postulates that the attentional resources from a previous race could be locked during a new race. In explaining results of a dwell time paradigm [79], the authors assume a ‘locking of resources’ during recoding of task-relevant features of VSTM objects into a non-visual format. TTVA ([56], p. 1031) introduces the ‘novel assumption that retention of a stimulus (e.g. T1) to be remembered in VSTM takes up visual-processing resources used to identify the stimulus. Until the stimulus is recoded into a nonvisual (e.g. auditory, motoric or amodal) format, the resources are locked and cannot be used to encode subsequent stimuli (e.g. T2) into VSTM. This mechanism creates a temporary encoding bottleneck that explains the time course of the AD (attentional dwell time). A difference between TTVA and TRAM is that TRAM assumes that retention of visual information for later report *per se* should not call a protective maintenance process with encapsulation and ‘resource locking’. According to TRAM, the process of *encapsulation* and ‘resource locking’ should only be triggered if phase 3 short-term consolidation for later report could not be finished when a new competition episode, that is, a *change of relative attentional weights*, and when an *object continuity failure* has been signalled.

### (c) Encapsulation of short-term consolidation during subsequent competition episodes. II. A new look at ‘rapid serial visual presentation’ and the ‘attentional blink’

As emphasized in §2, such non-clearance and encapsulation should have substantial attentional costs in terms of occupied slots and retained attentional weights. Highly informative in terms of understanding and specifying the processes behind these attentional costs are findings from the AB paradigm. In the following, a new look will be made at several key findings of the AB paradigm: the SOA-dependent core T2 deficit at short SOAs that recovers over time (the AB proper), lag-one sparing (including sparing for up to three Ts), the effect of a short post-T1 blank (150 ms) in eliminating the AB, as well as the reappearance of an AB within increasing SOA between T1 and T2 without any intervening D (lag-one sparing).

The AB paradigm belongs to a class of experiments with rapid serial visual presentation (RSVP). An RSVP stream consists of one target T stimulus among several distractor (D) stimuli. Every stimulus appears at the same location. At the end of the stimulus sequence of a trial, the T stimulus has to be reported without time pressure [80]. The findings show that even for fast presentation rates of roughly 100 ms, T report reaches a high performance level. How does TRAM describe the processing dynamics within an RSVP situation? The presentation rate of items within an RSVP stream (e.g. 100 ms) is sufficient for encoding each item within VWM—otherwise, the report of a single T would be hard to explain [80]. A D followed by a D should lead neither to a relative weight change and a new competition episode nor to a failure of object continuity owing to priority map region changes (e.g. attentional weight, location or rough shape). Each D re-categorizes the preceding D with a new feature (visual category) encoded into the same VWM slot. The competition episode remains the same as long as Ds are encoded. However, a change of the current competition episode occurs *if a D is followed by a T*. Clearly, this D–T sequence is accompanied by a change of attentional weights, that is, the D has a substantially lower weight than the T. Therefore, a new competition episode is called by the T and as a consequence, the preceding D is cleared from VWM. Ds are usually not subject to phase 3 operations and protective maintenance processes. During the new T competition episode, the features of the T-proto-objects should be encoded into VWM. After VWM encoding of the T, its phase 3 operations, that is, short-term consolidation for later report, starts. The *D directly after the T* signals an object continuity failure due to local object-specific weight change and again a new competition episode owing to a global change of relative attentional weights. Consequently, a clearance signal for VWM should be issued. Because the T is still subject to an ongoing phase 3 operation, namely short-term consolidation, 100 ms are not sufficient to complete all three processing phases including short-term consolidation. The T will be spared from VWM clearing by the protective maintenance process. Further trailing Ds will have to compete for VWM access with the encapsulated T as long as its short-term consolidation process is going on.

A standard AB experiment consists of an RSVP stream within two Ts (T1 and T2) within a stream of Ds [77]. The presentation time for each item is usually fixed (e.g. 100 ms). In a typical experiment, two letters appear within a stream of digits [76]. A deficit in reporting T2, called the AB ([74], see also [81]), emerges if the SOA between T1 and T2 is short and, usually, if Ds appear in between the Ts [82]; but see, findings of Nieuwenstein *et al*. [83], discussed in the last paragraph of this subsection. Importantly, as the SOA between T1 and T2 becomes larger and more Ds intervene, the AB becomes weaker until the T2 deficit disappears at long SOAs—usually, the disappearance occurs at SOAs of 500–800 ms [77].

First, why does the *core SOA-dependent T2 deficit*, the AB, emerge at all? The first phase of a trial of an AB experiment—successive Ds until the appearance of T1—has already been described above in the RSVP section. Crucially, the trailing D after T1 causes an attentional weight change that issues a new competition episode for the D. The new competition episode of the D calls for clearance of VWM. However, T1 with its still ongoing phase 3 operation of short-term consolidation is spared from clearance by the protective maintenance process and the T trailing D signals a failure of object continuity. Every subsequent stimulus—D or T—will suffer from the encapsulated T1, from its attentional weight, as long as short-term consolidation—a necessary condition for protection—is going on. If *T2 appears after a D*, then a new competition episode will be started. A change in terms of attentional weights from the preceding D to T2 occurs. Therefore, T2 competes with T1 during its phase 2-based attempt to access VWM. More precisely, as long as *phase 3 operation of short-term consolidation for T1 is going on, encapsulated T1 competes with T2 during phase 2 of its competition episode.* Decisive for competition is the maintained attentional weight of T1 that is linked to its VWM object representation. In other words, as long as short-term consolidation for T1 is working, its corresponding attentional weight of the priority map region is encapsulated and competes with the weight of T2. The simultaneous presence of the T1 weight with the T2 weight during the T2 competition episode slows the race of T2 towards VWM (TVA) down considerably. Why does a D or pattern mask (interruption masking) after T2 lead to the emergence of an AB? [77] Without a mask, iconic memory of T2 allows its VWM access despite its much slower ‘race speed’. In short, as long as short-term consolidation for T1 is going on, the chance of T2 to win the competition for VWM access should be substantially reduced by coexisting T1. If T2 is not able to access VWM prior to the appearance of a subsequent D, then T2 will be cleared by the D competition episode—as suggested by classical two-phase-based resource depletion theories of the AB [76]. Protective maintenance for an object is possible only after VWM encoding.

Why does *T2 performance improve with SOA*? The longer the SOA, the higher the chance that phase 3's short-consolidation of T1 has been already finished so that T1 will not be subject anymore to protective maintenance and encapsulation. If T1 is not protectively maintained, then the trailing item (D or T2) will call a new competition episode and clear the unprotected T1 from VWM. In this case, T1 should not be able to compete anymore with T2. The longer the SOA between T1 and T2, the higher the chance of a finished short-term consolidation for T1 in phase 3, and the less likely the chance of a competition between T1 and T2 and the less pronounced the T2 deficit should be. More formalized and as suggested by Jolicoeur & Dell'Acqua [69], short-term consolidation duration might follow an exponential distribution.

Second, this explanation of the SOA-dependent core T2 deficit is in line with the standard explanation of resource depletion theories [69,76]. However, it is a reductionist explanation of the ‘bottleneck effect’ in terms of weight-based competition between Ts and encapsulation. A major divergence between TRAM and classical two-stage theories of the AB emerges if a second key finding of the AB has to be explained, namely *lag-one sparing* (e.g. D, T1, T2, D), or sparing for several consecutive Ts (e.g. T1, T2, T3; [84,85]). In all these cases, no AB for T2 or T3 can be observed. The standard two-phase-based resource depletion explanations of the AB mentioned above have to introduce further assumptions for explaining this surprising finding. For TRAM, sparing of the AB is a direct consequence of the postulated processing architecture. Each T that directly follows a preceding T (e.g. T2 follows T1) without an intervening D does not call a new competition episode. T1 and T2 usually do not differ in terms of their attentional weights. Therefore, a T re-categorizes the preceding T and no change is signalled in terms of relative attentional weights. Therefore, a T that is followed by another T with the same attentional weight is not subject to protective maintenance and encapsulation. Instead, competition-free re-categorization and encapsulation of Ts takes place. Re-categorization should not interrupt short-term consolidation of the preceding categorization as part of one competition episode. Protective maintenance is called and an object continuity failure signalled by the first D after a series of Ts. It will encapsulate all categorizations and allows them to finish despite new trailing items. This explanation predicts that lag-one sparing should disappear if the presentation of T2 is accompanied by an attentional weight change.

Third, a *blank after T1* of a sufficient duration (100–150 ms) reduces or even eliminates the T2 deficit significantly [75]. This finding is highly important because it falsifies all explanations of the AB that assume a capacity-limited operation for T1 (such as short-term consolidation) of a fixed duration within the AB range (e.g. 500–800 ms) as the core of the T2 deficit [76]. The devastating effect of encoding T1 into VWM seems to depend on the immediate availability of a trailing item. How does TRAM explain this important finding? After an additional blank of 100 ms after the end of T1 presentation (also usually approx. 100 ms) leads to iconic decay of the features and weights of T1. If the D appears next, then it will signal an object continuity failure and encapsulates T1 at a certain level of iconic activation at the time of the D appearance, or more precisely, at the time of a call of encapsulation by the D. Therefore, after 100 ms blank, T1 has undergone iconic decay and it will thereby be encapsulated with a lower weight compared with a condition with an immediately following D. Consequently, T1 is a weaker competitor for T2, and the chance of VWM access of T2 should be substantially increased, that is, the AB should be reduced or even eliminated.

Fourth, this suggestion that an iconically decayed T1 weight is a weaker competitor for T2—compared with standard RSVP conditions—will now be applied in explaining a recently published finding of the *nature of lag-one sparing* by Nieuwenstein *et al.* [83]—a finding that appears counterintuitive and puzzling for almost all published AB theories. The authors manipulated the SOA between T1 and T2 in a lag-one sparing situation without any intervening D. In addition, they shortened the presentation of a masked T2 considerably (50 ms instead of 100 ms). Surprisingly, a classical AB curve with a large T2 deficit was observed in this condition of reduced and efficiently backward masked T2 presentation time. More precisely, if T2 follows T1 directly with small blank (50 ms item presentation followed by a 50 ms blank), then lag-one sparing is observed again. If the blank interval after T1 disappearance and T2 onset increases to 150 and 250 ms, then a large drop in T2 performance was found that recovered continuously during 500 ms. How does TRAM explain these findings? If T2 follows T1 directly, then no substantial iconic decay occurs, and no change of attentional weights is signalled. Therefore, no new competition episode, no protective maintenance process and encapsulation are called. A blank of about 150 ms and more after the end of T1 presentation leads to substantial iconic decay of the T1 weight. Iconic decay should reduce the absolute and also the relative weight of T1 (given that not just T1 but also other items of the current fixation such as the screen frame receive an attentional weight within its episode—an assumption that seems rather likely). Therefore, a change in attentional weights between decayed T1 and T2 is signalled. Consequently, a new competition episode, protective maintenance and encapsulation for T1 are called. The encapsulated weight of T1 is in this experiment a strong competitor for T2 given that the T2 presentation duration has been substantially shortened (from 100 to 50 ms). Therefore, a strong AB is observed. The longer the interval between T1 end and T2 appearance, the stronger will be the iconic decay of T1 and therefore the weaker will be the competition effect of T1 on T2. Therefore, the size of the AB should decrease with an increasing interval between the two Ts.

What is the major difference of TRAM's explanation of the AB and other explanations? [69,76,82,86] In short, TRAM offers a reductionist explanation of the T2 deficit described as a bottleneck phenomenon with strong deficits at short SOAs that recover with increasing SOA. It is claimed that an unfinished phase operation of short-term consolidation that started during the preceding competition episode binds and encapsulates attentional weights during the current competition episode of T2 access to active VWM. The degree of competition between the encapsulated T1 weight and the T2 weight determine the chance of VWM access of T2. Necessary for the call of encapsulation of T1 should be a change of attentional weights from T1 to the immediately trailing item (object continuity failure)—an assumption that is not shared by any other AB theory and that should imply unique predictions.

## 4. Implications of task-driven visual attention and working memory: a selective look at the interaction of visual working memory and visual search tasks

Here, a few selected implications of TRAM for explaining the enormous and still nonlinearly growing experimental literature on the interaction of visual attention (especially visual search) and working memory (for overviews, see [36,65]) should be spelled out. TRAM's assumption that VWM should be cleared within a new competition episode relatively late, at the end of phase 1, will be the explanatory key. This property is not only necessary for understanding the cross-episode interference effects such as AB effects of two consecutive targets without intervening distractors (reported by Nieuwenstein *et al*. [83], see the preceding section). It also allows a new look at biasing of visual search by trial-wise set-up VWM search templates. Moreover, based on the assumption of late clearance, results from dual task paradigms with ‘visual search during VWM retention’ will be analysed. It will be claimed that the presence and absence of interference effects of retained VWM objects on visual search should be caused by the clearance or non-clearance of the activation-based VWM retention objects prior to visual search.

### (a) Biasing competitive visual processing by trialwise visual search templates

In most visual search experiments, the search target (e.g. ‘search for black letter X!’) is constant within a block of trials and sometimes even across the complete experiment [34]. For such visual search tasks, it has been claimed that a repeated search target allows acquiring of and using a long-term memory-template of the target [87]. By contrast, if the visual search target varies from trial to trial [88], then a VWM search template should bias competition in the search process [87]. This assumption of a search template within VWM is in line with many versions of the biased competition approach [2,20,21]. A still open question is how the search template might be retained in VWM and how its biasing effect might be realized mechanistically. TRAM suggests a non-trivial answer. First, the trialwise varying search target item is usually visually presented at the beginning of the trial [88]. TRAM implies for this case that the search target is encoded within activation-based VWM. The search display appears a few hundred milliseconds later. Importantly, the search target encoded at the beginning of the trial is not encapsulated in VWM anymore when the search display appears. Short-term consolidation of the search target has already been completed at search display appearance, so that no protective maintenance and encapsulation can be triggered. Second, how this biasing effect might be realized can be directly derived from TRAM's processing dynamics, especially its feature of late VWM clearance. As stated above, the search target is retained after encoding at the beginning of the trial until the end of phase 1 of the next competition episode. The appearance of the search display initiates the next competition episode. During processing phase 1 of the search display—the computation of the attentional weights of the display elements—the search template from the preceding competition episode is still present within VWM. Biasing of attentional weight computation takes place.

Given this explanation, an important question emerges: if the search target is retained in active VWM why does it not—instead of biasing the computation for sensory-derived weights of the new episode—bring in its attentional weight as a further element of weight computation? If this would be the case, then massive interference effects—see the AB explanations above—should be observed. Why are no inference costs observed? VWM objects encapsulated with ongoing phase 3 operations create interference effects, but not VWM objects without phase 3 operations or VWM objects after the end of phase 3. As stated above, in visual search experiments with trialwise varying search targets, phase 3 of the search target (STM consolidation) should be clearly finished by the time the search display appears. After finishing, iconic decay of features and the attentional weight of the search target should take place within the ongoing processing episode (see, above, the explanation of the findings of Nieuwenstein *et al*. [83]). However, even after the iconic decay of features and the weight, the VWM pointer of the search target should still be present (e.g. due to self-excitation, see [3]). The VWM pointer can only be eliminated by the clearance signal from the next episode at the end of phase 1. In other words, immediately prior to the onset of the search display, the search target should be present as an active representation of the VWM pointer, that is, as a pointer without retained features or features at a very low activation level, and therefore also without a retained priority map region. Biasing signals from VWM pointers but not retained objects should survive the end of phase 3 and iconic decay of the search template. Therefore, prior to VWM clearance, the visual search template, the pointer, should exert its biasing influence. Owing to short-term consolidation, the temporary connections of the pointer to the features (without or very low activation) still exist and allow top-down biasing. The clearance of the search template from active VWM prior to the start of competitive phase 2 has the advantage that all VWM slots are available. Visual search should be efficient ‘even if VWM is full’ (see [87]).

### (b) The presence and absence of interference effects in dual task paradigms with visual search during visual working memory retention

Olivers *et al*. [65] offer a review on the interaction of VWM tasks with visual search tasks. In many of these reviewed dual tasks, a search process (task 2) has to be carried out, whereas items have to be retained in VWM (task 1) for a later response (e.g. change detection or recognition). The findings from the dual task experiments are complex, showing sometimes effects of retained VWM items on visual search performance, and sometimes no effects. For instance, the Olivers *et al*.'s [89] study, on the one hand, reported an effect of a VWM item on search. A coloured circle was presented at the start of the trial and had to be retained for later recognition (task 1). During the retention interval, a visual search task (task 2) was performed that contained a singleton distractor. If the colour of the singleton distractor matched the retained memory item, then visual search time was increased compared with a condition of non-match. This result is implied by the processing dynamics of TRAM. During the presentation of the coloured memory item at the start of the trial, its encoding into VWM takes place. Next, short-term consolidation of coloured task 1 item is initiated creating passive VWM traces for later retrieval and recognition. Given the long presentation duration of the task 1 item and an empty interval before the search display, consolidation of this item should clearly be finished by the time the search display appears. Before VWM clearance of the search target and during weight computation at the beginning of phase 1, the task 1 memory item exerts—similar to a trial-wise search template—a biasing effect by increasing the attentional weight of the singleton distractor. The VWM pointer of the search target is still active (prior to VWM clearance) and exerts after the end of short-term consolidation nevertheless an effect via its temporary connections to features. The fact that short-term consolidation has ended does not imply that activation of VWM pointer should be reduced to baseline [3]. As stated above, an activated pointer within the VWM map of objects can be eliminated only by the VWM clearance process. Consequently, owing to biasing by the VWM pointer of the search target, search time increases compared with a non-matching singleton that did not (or to a weaker degree) receive a biasing signal from the VWM item.

On the other hand, the dual task study of Downing & Dodds [90] and other dual task studies (see [65]) reported no effect of visual short-term retention on visual search. In the Downing & Dodds [90] experiment, at the start of the trial, two items were presented, namely the STM item for later change detection (task 1), and the search target item (task 2) that varied trialwise. An efficient strategy would be, in this case, to encode the STM item first (competition episode *n*) and consolidate it into passive VWM for later comparison in the memory test phase. In the next step, competition episode *n* + 1 with a new attentional set (‘attend to the search target’), the search target item should be encoded into activation-based VWM. This step clears at the end of its phase 1 the STM item from episode *n* from active VWM. This order of competition episodes and VWM encoding allows the search target to bias the search process by a powerful activation-based code and it later allows a memory test based on passive VWM traces. Given this chain of events, the memory item should exert no biasing effect in visual search in competition episode *n* + 2. When the search display appears, the search target template encoded in episode *n* + 1 is still part of active VWM, whereas the STM item from episode *n* has already been cleared during episode *n* + 1. Therefore, no effect of the STM item should be observed.

This selective interpretation of two studies on interaction of VWM retention and visual search should illustrate the suggested processing dynamics of TRAM. However, more published studies and results [36,65] have to be analysed, and direct experimental tests are required before more firm conclusions about the explanatory capabilities of TRAM can be made.
